# Machine learning approaches to identify the link between heavy metal exposure and ischemic stroke using the US NHANES data from 2003 to 2018

**DOI:** 10.3389/fpubh.2024.1388257

**Published:** 2024-09-16

**Authors:** Yierpan Zibibula, Gulifeire Tayier, Aierpati Maimaiti, Tianze Liu, Jinshuai Lu

**Affiliations:** ^1^Department of Emergency Center II, People's Hospital of Xinjiang Uygur Autonomous Region, Ürümqi, Xinjiang, China; ^2^Department of Critical Care Medicine, The First Affiliated Hospital of Xinjiang Medical University, Ürümqi, Xinjiang, China; ^3^Department of Neurosurgery, Neurosurgery Centre, The First Affiliated Hospital of Xinjiang Medical University, Ürümqi, Xinjiang, China

**Keywords:** machine learning, ischemic stroke, heavy metals exposure, NHANES, logistic regression

## Abstract

**Purpose:**

There is limited understanding of the link between exposure to heavy metals and ischemic stroke (IS). This research aimed to develop efficient and interpretable machine learning (ML) models to associate the relationship between exposure to heavy metals and IS.

**Methods:**

The data of this research were obtained from the National Health and Nutrition Examination Survey (US NHANES, 2003–2018) database. Seven ML models were used to identify IS caused by exposure to heavy metals. To assess the strength of the models, we employed 10-fold cross-validation, the area under the curve (AUC), F1 scores, Brier scores, Matthews correlation coefficient (MCC), precision-recall (PR) curves, and decision curve analysis (DCA) curves. Following these tests, the best-performing model was selected. Finally, the DALEX package was used for feature explanation and decision-making visualization.

**Results:**

A total of 15,575 participants were involved in this study. The best-performing ML models, which included logistic regression (LR) (AUC: 0.796) and XGBoost (AUC: 0.789), were selected. The DALEX package revealed that age, total mercury in blood, poverty-to-income ratio (PIR), and cadmium were the most significant contributors to IS in the logistic regression and XGBoost models.

**Conclusion:**

The logistic regression and XGBoost models showed high efficiency, accuracy, and robustness in identifying associations between heavy metal exposure and IS in NHANES 2003–2018 participants.

## 1 Introduction

This study focuses on ischemic stroke (IS), which is a major global health issue. Strokes can be classified as hemorrhagic stroke (HS) and ischemic stroke (IS). IS accounts for 87% of stroke cases (1) and has become a critical disease burden globally. Researchers have found that chronic hypertension (2), diabetes (3), the age of menopause (4), dyslipidemia, obesity, and smoking (5) are the risk factors for IS. However, there is limited research on the relationship between heavy metals and IS (6–8).

Heavy metals, such as lead, mercury, and cadmium, are considered some of the most hazardous risk factors due to their non-biodegradable nature (9). They can accumulate in the human body, exert neurotoxic effects, and increase the risk of cardiovascular diseases. Therefore, investigating the association between heavy metal exposure and ischemic stroke is significant.

Previous studies, relying on traditional statistical methods and often focusing on single-exposure models, had limitations in capturing the non-linear relationships and complex interactions between multiple heavy metals and IS risk, which are crucial for understanding the multifaceted nature of heavy metal toxicity. In the field of ML, computers leverage algorithms to learn from and discern patterns in data, offering robust computational and data-fitting abilities for uncovering complex data relationships, which is a common practice in clinical studies (10). Moreover, ML models are instrumental in identifying hazards, assisting in disease diagnosis, and facilitating health-related decisions (11).

In this research, we used the National Health and Nutrition Examination Survey (US NHANES, 2003–2018) datasets to explore the relationship between IS and heavy metal exposure. Seven ML models were constructed, and then the DALEX package was utilized to investigate the contribution of each heavy metal to the identification of IS, enhancing the potential for early intervention.

## 2 Materials and methods

### 2.1 Study population

The NHANES is a multi-stage, stratified, large-scale, and nationally representative study of the US population. It aims to assess the nutrition and physical condition of Americans. The data of the research participants included eight contiguous cycles from 2003 to 2018 of the US NHANES.

The inclusion criteria were as follows: (1) the participants were 20 years or older; (2) the participant information about ischemic stroke (IS) status was verified using the data from the US NHANES questionnaires; and (3) the participants were involved in the sub-study that focused on the analysis of heavy metals in urine and blood. The exclusion criteria were as follows: (1) the participants who had missing data for more than two heavy metals in the dataset; (2) the participants who exhibited indeterminate ischemic stroke (IS) status based on the US NHANES questionnaires; and (3) the samples that could not be adapted to the model for any reason. These criteria ensured that our analysis was based on comprehensive and reliable data, which minimized the impact of incomplete records on the study's findings.

### 2.2 Feature extraction and preprocessing

The participants' demographic and socioeconomic characteristics were collected from the questionnaire data of the NHANES. The characteristics included age, marital, race/Hispanic ethnicity, sex, body mass index (BMI, kg/m^2^), education level, and poverty-to-income ratio (PIR).

The concentrations of heavy metals, including cadmium, lead, and total mercury in blood and antimony, barium, cadmium, cobalt, cesium, lead, molybdenum, thallium, tungsten, uranium, total arsenic acid, arsenobetaine, monomethyl arsenic acid, dimethylarsenic acid, and mercury in urine, were included for analysis. Heavy metal exposure levels were measured using the inductively coupled plasma dynamic reaction cell mass spectrometry (ICP-DRC MS), following comprehensive quality procedures established by the National Center for Environmental Health.

The preprocessing steps included the following:

Handling of missing data:

Participants with missing data for more than two heavy metals were excluded from the analysis to ensure data completeness. For participants with fewer missing values, we employed multiple imputation techniques to manage the missing data, ensuring that the missingness did not introduce bias into the models.

Encoding of categorical variables:

Categorical variables, such as marital status, race/Hispanic ethnicity, and education level, were encoded using one-hot encoding to create binary indicators for each category. This method prevented the model from assuming any ordinal relationship between the categories.

Feature scaling:

Continuous variables, including heavy metal concentrations and BMI, were standardized using Z-score normalization. This step ensured that all features had a mean of 0 and a standard deviation of 1, which is crucial for the performance of distance-based models, such as k-nearest neighbors (kNN) and support vector machines (SVM). Feature scaling was applied after the data were split into training and testing sets to prevent data leakage.

Outlier detection and treatment:

Outliers in the heavy metal concentration data were identified using the interquartile range (IQR) method. The values that fell outside 1.5 times the IQR above the third quartile or below the first quartile were considered outliers. These outliers were either capped at the nearest non-outlier value or retained, depending on their impact on the model performance during cross-validation.

Feature selection:

To reduce dimensionality and prevent overfitting, we applied feature selection techniques, such as recursive feature elimination (RFE) and feature importance from tree-based models (12), to identify the most relevant predictors of IS. This step was performed within the training set to ensure that the testing set remained unseen during the model training.

### 2.3 ML model strategies

The participants were divided into training and testing sets in a ratio of 7:3. IS caused by heavy metal exposure was identified using the ML models, which included decision tree (DT), logistic regression (LR), LightGBM, random forest (RF), XGBoost, k nearest neighbors (kNN), and support vector machine (SVM). A brief overview of each technique is provided as follows: DT: A model that splits the data into branches to make predictions and is known for its simplicity and interpretability but is prone to overfitting (13). LR: A linear model that is used for binary classification that estimates probabilities and is known for its simplicity and efficiency but assumes linear relationships (13). LightGBM: A gradient boosting framework that uses tree-based learning algorithms and is known for its high efficiency and scalability but can be sensitive to overfitting (14). RF: An ensemble method that builds multiple decision trees and merges them to improve accuracy and is known for its robustness but can be computationally intensive (13). XGBoost: An optimized gradient boosting algorithm known for its high predictive performance but requires careful parameter tuning (13). kNN: A simple, instance-based learning algorithm that classifies data points based on their proximity to neighbors and is known for its simplicity but can be computationally expensive with large datasets (13). SVM: A model that identifies the hyperplane that best separates the classes and is known for its effectiveness in high-dimensional spaces but can be less interpretable (13). The discrimination of the ML models was shown by the area under the curve (AUC), F1 scores, decision curve analysis (DCA) curves, Matthews correlation coefficient (MCC), precision-recall curves, and Brier scores.

### 2.4 Statistical analysis

The categorical variables were presented as numbers (%), while the continuous variables were presented as medians (quartile ranges) or geometric means ± standard deviations. The chi-squared test or Wilcoxon two-sample test was performed to compare the characteristics of the different groups. All analyses were performed using R software (version 4.0.2), and a *p* < 0.05 was considered statistically significant.

## 3 Results

### 3.1 Demographic characteristics

The characteristics of the study participants are shown in Table 1. A total of 15,575 participants were included in this research. The patients were divided into IS and non-IS groups based on whether IS occurred during their hospital stay. Among them, 622 cases were diagnosed with IS. The average age of the IS patients was 65 years, and 52.4% of them were male patients. The participants with IS were more likely to be older, married, non-Hispanic white, with higher BMI, high school graduate/GED or equivalent, and with a lower PIR (all *p* < 0.05).

**Table 1:** Characteristics of the study participants.

**Name**		**Non-IS**	**IS**	***p***
		**(*****N*** = **14,953)**	**(*****N*** = **622)**	
Age	Mean ± SD	48.6 ± 17.6	65.0± 13.5	< 0.001
Marital	Divorced	1,597 (10.7%)	105 (16.9%)	< 0.001
	Living with partner	1,334 (8.9%)	27 (4.3%)	
	Married	7,554 (50.5%)	309 (49.7%)	
	Never married	2,816 (18.8%)	50 (8%)	
	Separated	534 (3.6%)	20 (3.2%)	
	Widowed	1,118 (7.5%)	111 (17.8%)	
Eth	Mexican American	2,311 (15.5%)	56 (9%)	< 0.001
	Non-Hispanic Black	3,277 (21.9%)	180 (28.9%)	
	Non-Hispanic White	6,419 (42.9%)	304 (48.9%)	
	Other Hispanic	1,344 (9%)	35 (5.6%)	
	Other race—including multi-racial	1,602 (10.7%)	47 (7.6%)	
Sex	Female	7,517 (50.3%)	296 (47.6%)	0.204
	Male	7,436 (49.7%)	326 (52.4%)	
BMI	Mean ± SD	28.9 ± 6.9	29.5 ± 6.7	0.031
Education	9–11th Grade (Includes 12th grade with no diploma)	2,223 (14.9%)	125 (20.1%)	0 < .001
	College Graduate or above	3,100 (20.7%)	75 (12.1%)	
	High school graduate/GED or equivalent	3,546 (23.7%)	166 (26.7%)	
	Less than 9th grade	1,624 (10.9%)	102 (16.4%)	
	Some college or AA degree	4,460 (29.8%)	154 (24.8%)	
PIR	Mean ± SD	2.4 ± 1.6	2.0 ± 1.4	< 0.001

### 3.2 Concentrations of heavy metals

The concentrations of heavy metals in urine and blood for each data release cycle are shown in Table 2. The results revealed that cadmium, lead, and total mercury in blood and antimony, barium, cadmium, cobalt, cesium, lead, molybdenum, thallium, tungsten, total arsenic acid, arsenobetaine, monomethyl arsenic acid, dimethylarsenic acid, and mercury in urine showed significant tendencies (all *p* < 0.05).

**Table 2:** Geometric means and geometric standard deviations of heavy metals by each cycle.

	**2003–2004 (*N* = 1,540)**	**2005–2006 (*N* = 1,516)**	**2007–2008 (*N* = 1,854)**	**2009–2010 (*N* = 2,018)**	**2011–2012 (*N* = 2,271)**	**2013–2014 (*N* = 2,391)**	**2015–2016 (*N* = 2,282)**	**2017–2018 (*N* = 1,703)**	***p***
**Blood**									
Cadmium	0.6± 0.6	0.6± 0.6	0.6± 0.6	0.5± 0.6	0.7± 0.8	0.6± 0.6	0.6± 0.7	0.5± 0.6	< 0.001
Lead	2.1± 1.7	1.9± 1.5	1.9± 1.8	1.7± 1.7	1.7± 2.0	1.4± 1.5	1.4± 1.3	1.3± 1.2	< 0.001
Total mercury	1.5± 1.9	1.6± 2.0	1.5± 2.2	1.7± 2.6	1.6± 2.8	1.5± 2.7	1.5± 2.4	1.5± 3.2	0.038
**Urine**									
Antimony	0.1± 0.1	0.1± 0.2	0.1± 0.2	0.1± 0.1	0.1± 0.1	0.1± 0.2	0.1± 0.4	0.1± 0.2	< 0.001
Barium	2.1± 3.5	2.3± 3.9	2.1± 4.0	2.3± 9.9	1.9± 4.0	1.7± 2.8	1.9± 3.0	1.6± 2.3	< 0.001
Cadmium	0.5± 0.5	0.4± 0.5	0.4± 0.5	0.4± 0.5	0.4± 0.6	0.4± 0.4	0.4± 0.5	0.4± 0.5	< 0.001
Cobalt	0.5± 3.3	0.6± 1.6	0.5± 0.6	0.5± 1.2	0.5± 1.0	0.6± 1.1	0.6± 1.1	0.6± 1.4	0.019
Cesium	6.5± 17.4	5.8± 4.3	5.4± 4.2	5.0± 3.2	4.8± 3.3	4.9± 3.3	5.1± 4.2	5.2± 3.5	< 0.001
Lead	1.0± 1.0	1.0± 1.2	0.9± 1.6	0.8± 1.6	0.7± 1.2	0.5± 0.9	0.6± 0.7	0.5± 0.7	< 0.001
Molybdenum	55.1± 56.8	59.4± 51.9	60.6± 55.9	57.4± 55.4	52.7± 49.8	47.7± 51.8	50.2± 45.4	48.2± 47.0	< 0.001
Thallium	0.2± 0.1	0.2± 0.1	0.2± 0.1	0.2± 0.1	0.2± 0.1	0.2± 0.1	0.2± 0.2	0.2± 0.1	< 0.001
Tungsten	0.1± 0.2	0.1± 0.3	0.2± 0.3	0.1± 0.2	0.2± 0.8	0.1± 0.3	0.1± 0.2	0.1± 0.5	< 0.001
Uranium	0.0± 0.0	0.0± 0.0	0.0± 0.0	0.0± 0.0	0.0± 0.0	0.0± 0.0	0.0± 0.0	0.0± 0.0	0.333
Total; Arsenic	20.4± 52.2	24.4± 69.5	18.8± 51.5	24.5± 62.0	20.7± 55.8	15.0± 38.7	16.2± 35.0	19.9± 79.3	< 0.001
Arsenobetaine	9.9± 41.4	13.5± 56.0	8.6± 37.5	12.0± 42.0	12.0± 49.4	8.7± 35.4	9.4± 30.0	11.6± 68.7	0.004
Monomethylarsonicacid	1.0± 0.8	1.0± 1.2	1.0± 1.4	1.1± 4.2	0.9± 1.6	0.6± 0.7	0.6± 0.6	0.5± 0.6	< 0.001
Dimethylarsinicacid	5.5± 6.2	5.9± 9.5	5.6± 8.6	6.1± 9.2	6.3± 8.6	4.9± 5.9	5.1± 7.0	5.3± 8.4	< 0.001
Mercury	1.0± 2.9	0.9± 1.4	0.8± 1.3	0.7± 1.0	0.7± 1.8	0.6± 2.7	0.4± 0.9	0.4± 0.9	< 0.001

### 3.3 Construction and validation of ML models

**Figure 1:**
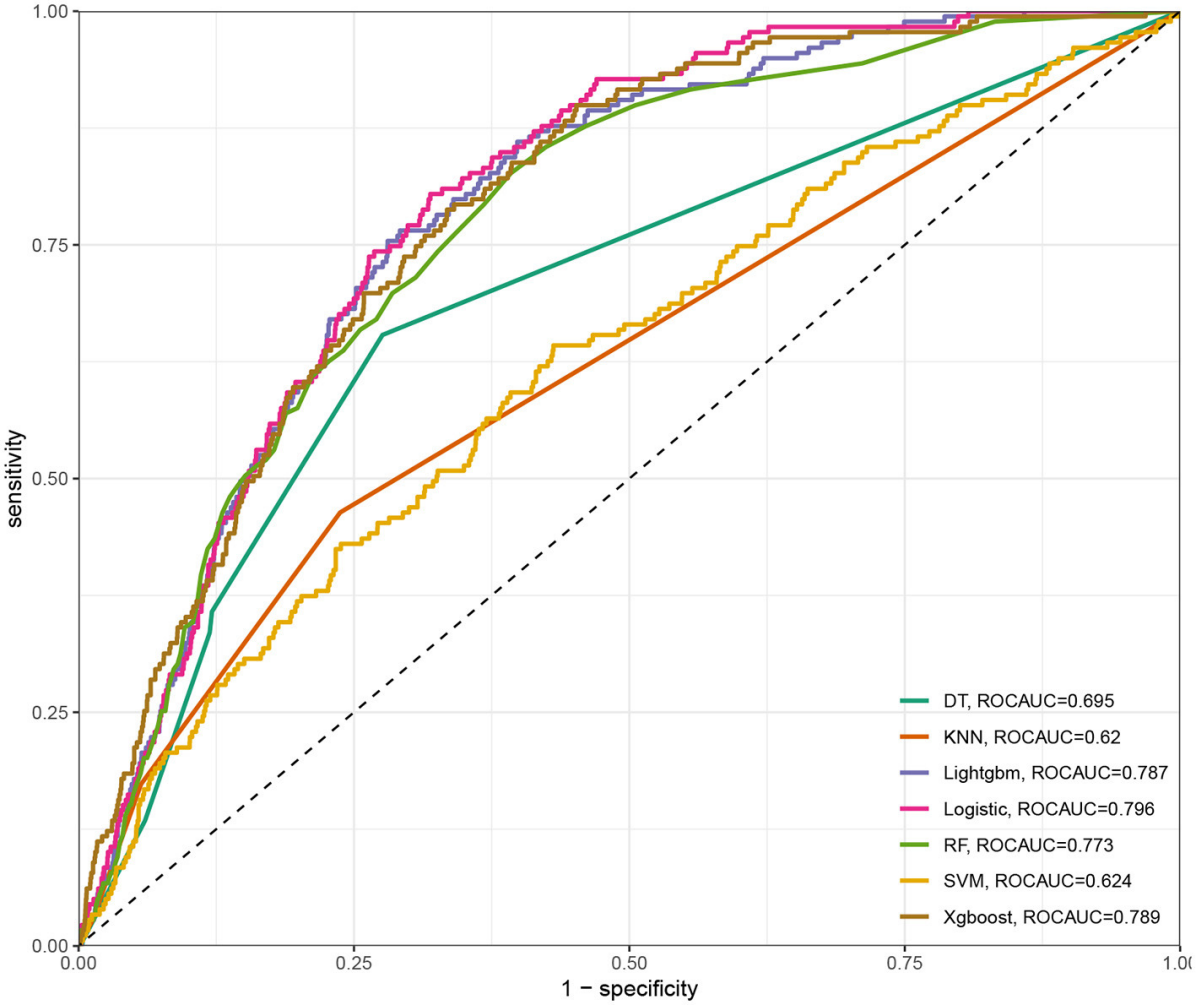
The area under the curve (AUC) and sensitivity-1-specificity curve for the seven ML models, including RF, XGBoost, LightGBM, DT, LR, kNN, and DT.

**Figure 2:**
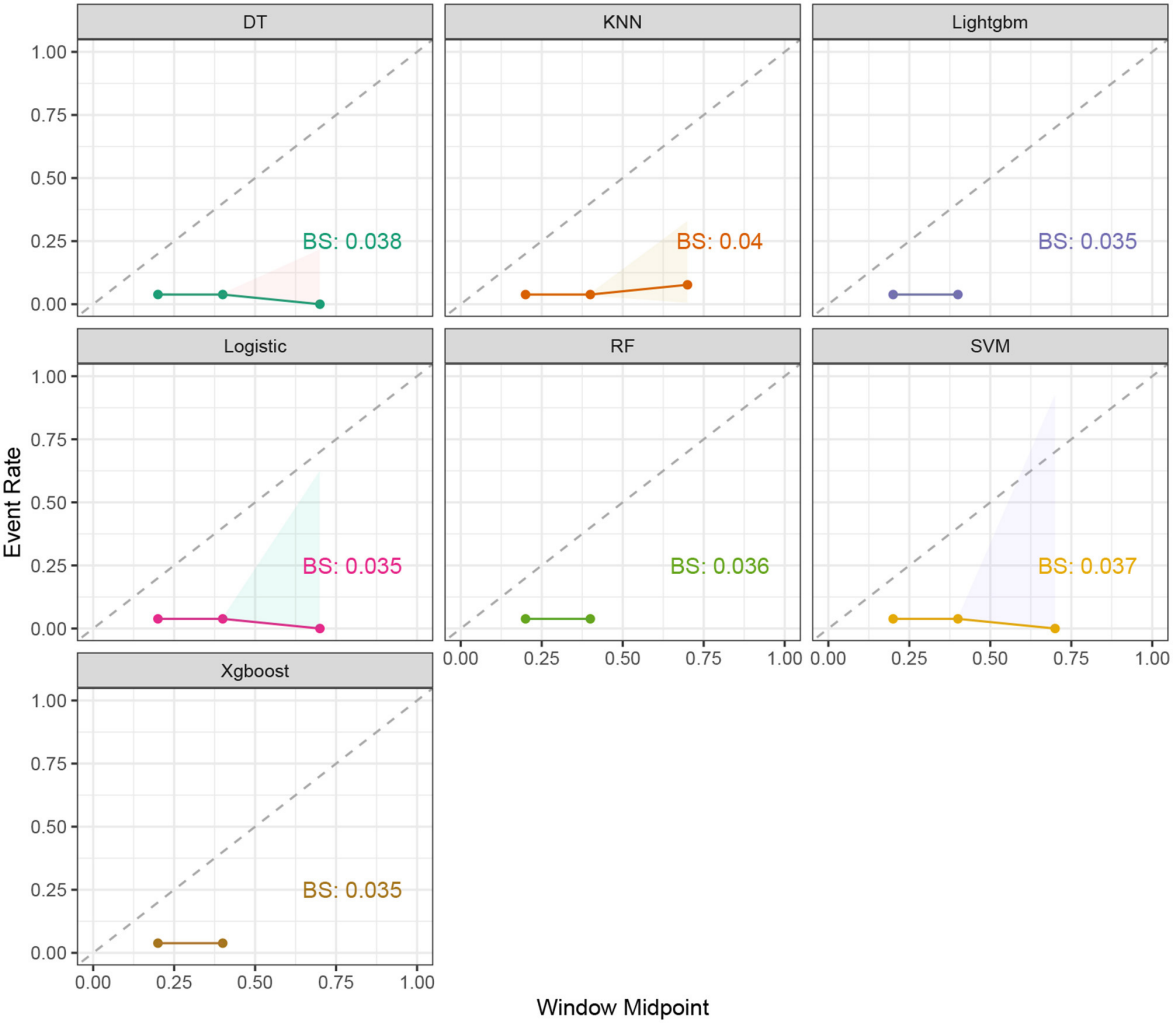
The event rate and window midpoint for the seven ML models, including RF, XGBoost, LightGBM, DT, LR, kNN, and DT.

**Figure 3:**
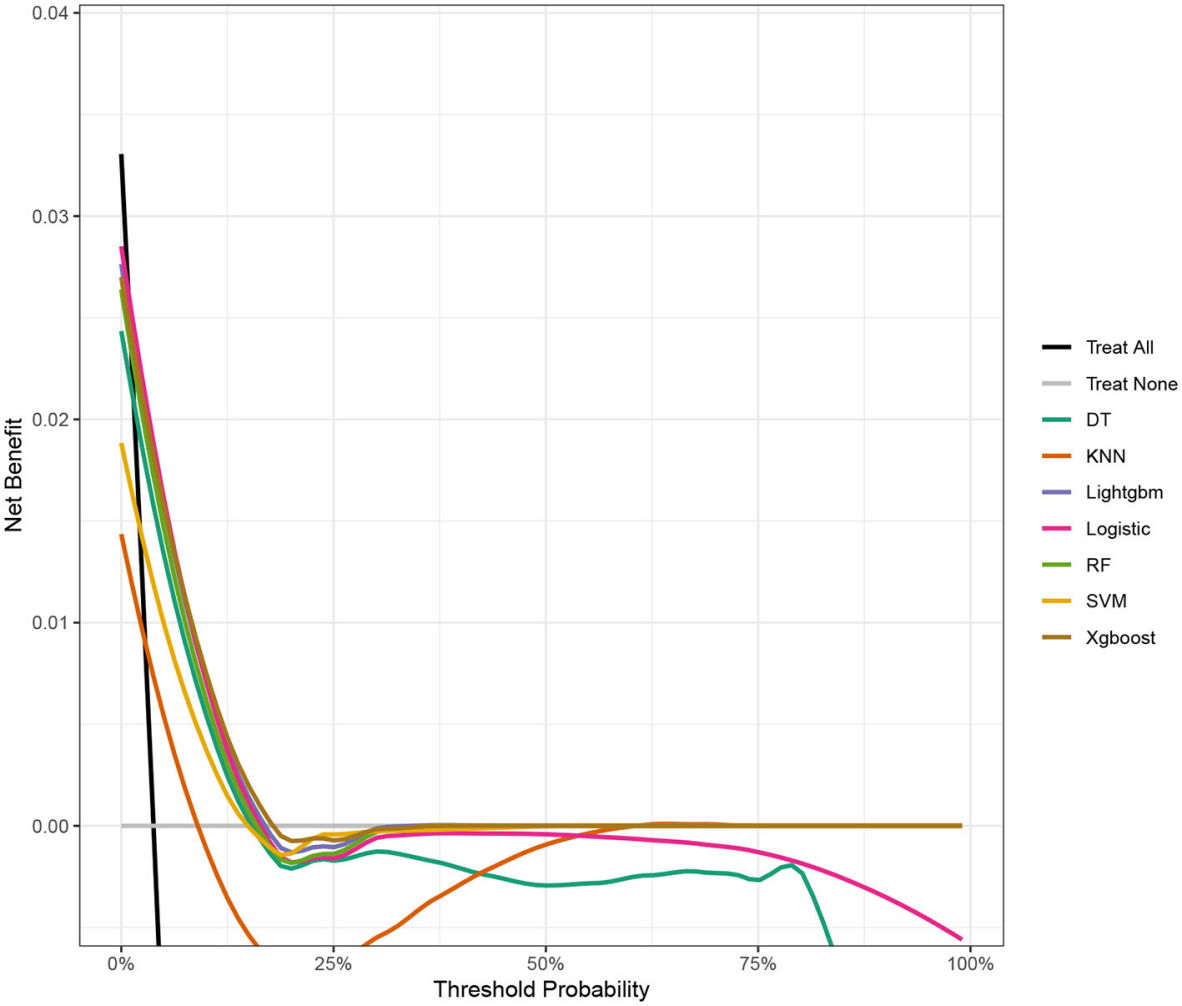
The DCA of the test data for the seven ML models, including RF, XGBoost, LightGBM, DT, LR, kNN, and DT.

**Figure 4:**
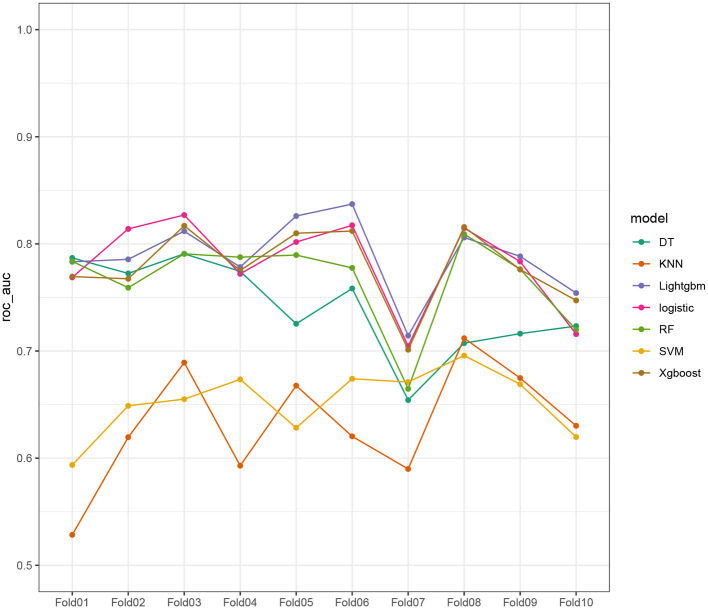
Demonstration of the 10-fold cross-validation tests.

Seven ML models, which included RF, XGBoost, LightGBM, DT, LR, kNN, and DT, were constructed. The AUC measures the ability of a model to distinguish between classes. An AUC of 1 indicates perfect discrimination, while an AUC of 0.5 suggests no discrimination (equivalent to random guessing) (15). As shown in Figure 1, the ROC analysis revealed that the LR model had the best AUC performance (AUC: 0.796), followed by XGBoost (AUC: 0.789), LightGBM (AUC: 0.787), and RF (AUC: 0.773), while DT (AUC: 0.695), SVM (AUC: 0.624), and kNN (AUC: 0.620) performed relatively poorer. However, given the imbalance between the positive and negative events in the dataset, the AUC alone was insufficient in fully assessing the performance of the models. To address the limitations of the ROC curve, we also generated a precision-recall (PR) curve, which provided a more informative evaluation under these conditions. As shown in Supplementary Figure 1, the PR curve clearly demonstrates that the logistic regression and XGBoost models achieved higher average precision compared to the other models. The F1 scores, Brier scores, and MCC of the XGBoost model were then assessed using a confusion matrix. The F1 score is the harmonic mean of precision and recall, providing a balance between the two. It is particularly useful when the class distribution is imbalanced. A higher F1 score indicates better model performance (16). As shown in Table 3, LightGBM (F1 score: 0.167) and XGBoost (F1 score: 0.155) demonstrate better predictive performance. The MCC is a robust metric that evaluates model performance by considering all four confusion matrix categories (true positives, true negatives, false positives, and false negatives), which makes it particularly useful for imbalanced datasets (17). As shown in Table 3, LightGBM (MCC: 0.193) and XGBoost (MCC: 0.178) had better predictive performance. The Brier score measures the accuracy of probabilistic predictions. It is calculated as the mean squared difference between predicted probabilities and actual outcomes. A lower Brier score indicates better model calibration (18). The Brier scores are shown in Figure 2, and the results revealed that the LR (Brier: 0.035), LightGBM (Brier: 0.035), and XGBoost (Brier: 0.035) models exhibit better predictive performance when compared with RF (Brier: 0.036), SVM(Brier: 0.037), DT (Brier: 0.038), and kNN (Brier: 0.04) models. DCA is used to evaluate the clinical utility of prediction models. It shows the net benefit of using a model across different threshold probabilities, aiding in determining a model's value in a clinical setting (19). The DCA curves show that the net benefits of the seven machine learning models were not much different. However, the accuracy of the logistic regression and XGBoost models was higher (Figure 3). Based on the 10-fold cross-validation test, the XGBoost and LR models performed better in predicting stroke associated with heavy metal exposure (Figure 4).

**Table 3:** Comparison of discrimination characteristics among seven ML models.

**ML model**	**F1 score**	**Sensitivity**	**Specificity**
Logistic	0.141754	0.871508	0.58478
DT	0.152344	0.653631	0.724077
KNN	0.124906	0.463687	0.762572
Lightgbm	0.16739	0.75419	0.710948
RF	0.141935	0.184358	0.943703
Xgboost	0.155074	0.759777	0.679795
SVM	0.104306	0.480447	0.692034

### 3.4 Visualization of feature importance

**Figure 5:**
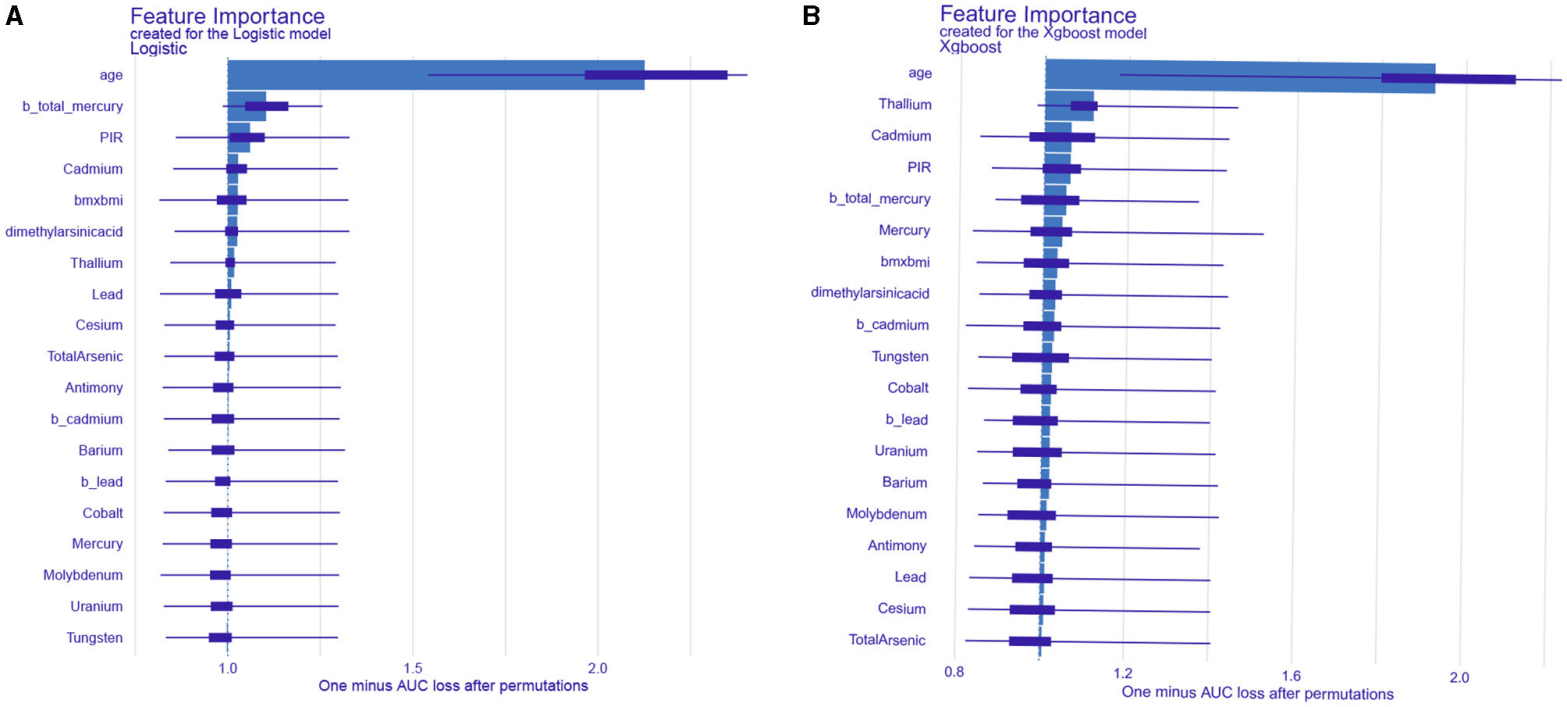
Analysis and visualization of the feature importance created for the logistic regression model **(A)** and XGBoost model **(B)**.

We calculated feature importance using the DALEX package, which provides an interpretable and reliable framework for understanding model predictions. The top 19 clinical variables were identified and ranked based on their importance in predicting the risk of ischemic stroke. As for LR, age was the most important factor, followed by total mercury in blood, PIR, cadmium in urine, and BMI (Figure 5A). In XGBoost, the first five key variables were age, thallium, cadmium, PIR, and total mercury in blood (Figure 5B).

**Figure 6:**
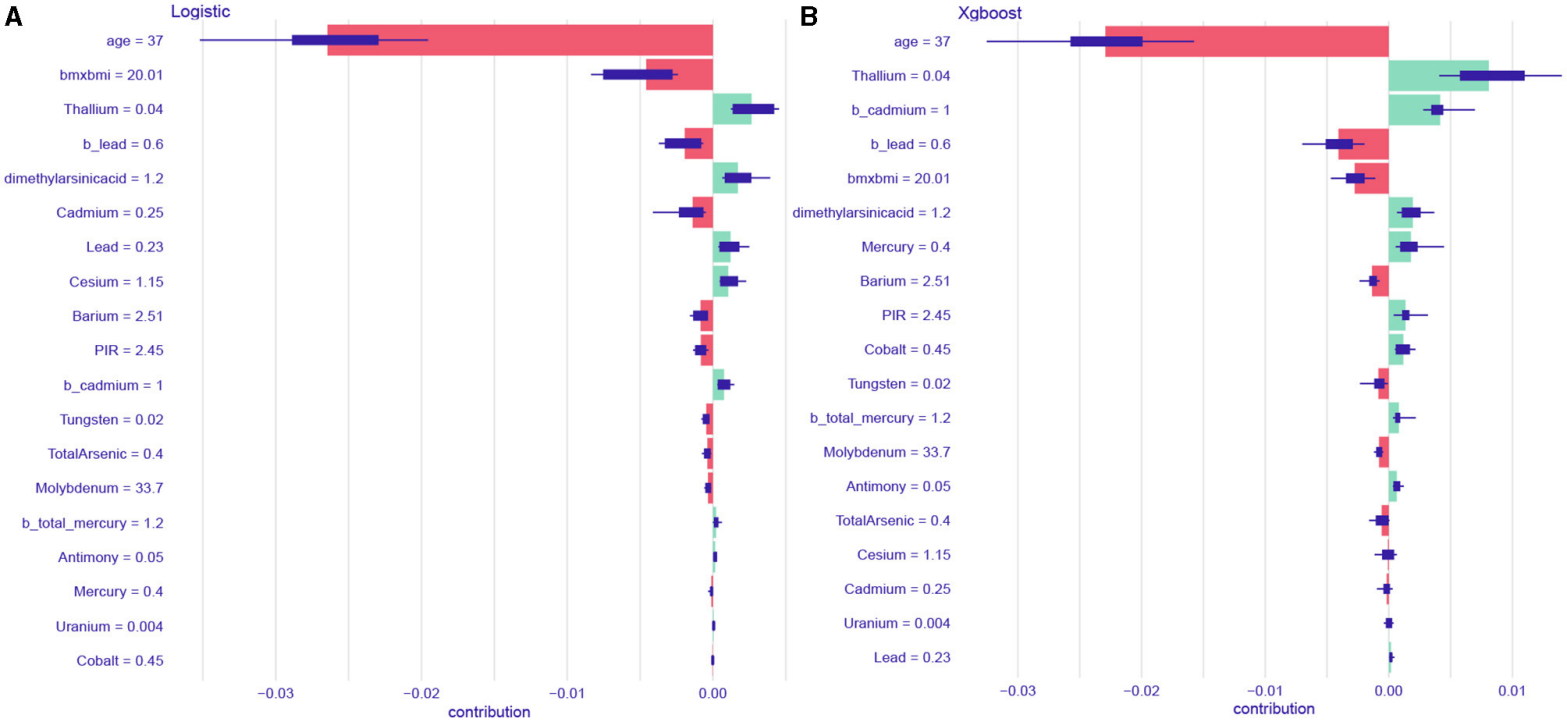
The single sample predictive decomposition diagram of the logistic regression model **(A)** and XGBoost model **(B)**.

A single-sample predictive decomposition diagram of the LR model showed that an age of 37 years, a blood lead level of 0.6 ng/mL, and a BMI score of 20.01 were the factors considered in the model. A urinary cadmium level of 0.25 ng/mL was identified as a protective factor for IS, indicating that it is associated with a lower risk of IS. Conversely, a urinary thallium level of 0.04 ng/mL and a dimethylarsenic acid level of 1.2 ng/mL were identified as risk factors for IS, indicating that they are associated with a higher risk of IS (Figure 6A). For the XGBoost model, an age of 37 years, a blood lead level of 0.6 ng/mL, and a BMI score of 20.01 were also considered. In this model, these factors were identified as protective against IS, while a blood thallium level of 0.04 ng/mL, a blood cadmium level of 1 ng/mL, and a dimethylarsenic acid level of 1.2 ng/mL were identified as risk factors for IS (Figure 6B). In summary, a specific combination of an age of 37 years, a blood lead level of 0.6 ng/mL, a BMI score of 20.01, and a urinary cadmium level of 0.25 ng/mL suggested that these factors were protective according to the LR model. However, other factors such as urinary thallium and dimethylarsenic acid levels still posed a risk. Similarly, for the XGBoost model, while age, blood lead, and BMI were the protective factors, the presence of blood thallium, blood cadmium, and dimethylarsenic acid levels were the risk factors. Therefore, while some factors (age, blood lead, BMI, and urinary cadmium) suggested a lower risk, the presence of other factors (urinary thallium and dimethylarsenic acid) indicated an increased risk. The overall risk for IS depends on the combined effect of all these factors.

## 4 Discussion

This large retrospective study explored the potential relationship between IS risk and exposure to 18 types of heavy metals by combining and analyzing the NHANES data from 2013 to 2018. Based on the ML models, we found that mercury in blood, PIR, and cadmium were significantly related to IS. In addition, using the DALEX package could illustrate the importance of the features in the model.

The data indicate that, annually, over 1,40,000 individuals succumb to stroke, making it the third leading cause of mortality in the United States (20, 21). Stroke represents the predominant type of cerebrovascular incident, with ischemic stroke (IS) being the most frequent, comprising approximately 85% of all stroke cases. The risk factors for IS can be divided into modifiable factors (diabetes, poor diet, hyperlipidemia, and high blood pressure) and immutable factors (genetics, age, and sex) (22, 23). Clinical strategies to manage modifiable risk factors can reduce the risk of IS.

The Agency for Toxic Substances and Disease Registry (ATSDR) has released a list of heavy metals that pose a considerable risk to human health (24). Arsenic, lead, cadmium, and mercury have been found to be associated with IS (7, 25). Studies have found that exposure to cadmium and lead increases the risk of IS by altering the endothelial function, increasing oxidative stress, downregulating nitric oxide production, promoting inflammation, and increasing the risk of peripheral artery diseases (26). As a trace element, mercury may be toxic to humans upon contact, and this exposure has been significantly associated with IS (23, 27). Humans do not possess the capability to efficiently eliminate mercury from their bodies (28). Prolonged exposure to mercury is known to lead to endothelial dysfunction, thereby elevating the risk of cardiovascular and cerebrovascular diseases (29). Consuming fish rich in Omega-3 oils can help reduce cardiovascular harm, including the risk of stroke, which is caused by exposure to mercury (30). Studies from the USA, Finland, and the UK have demonstrated a notable correlation between the levels of arsenic in drinking water and the incidence of cerebrovascular diseases, indicating a significant dose–response relationship (31). Furthermore, it has been found that blood lead is a positive risk factor for IS (32). However, the mechanism of lead in IS is still unclear (33). Barium and lead are known to penetrate the blood–brain barrier and impact brain tissue (34) and have been identified as significant risk factors for IS (6). A cigarette contains various heavy metals, including cadmium, arsenic, and lead (35). Therefore, smoking is also a risk factor for IS. The half-life of cadmium ranges from 1 to 30 years (36). An epidemiological investigation revealed that cadmium is a risk factor for atherosclerosis (37). Furthermore, an analysis of the data from the National Health and Nutrition Examination Survey revealed a positive correlation between the occurrence of stroke and the levels of cadmium (38, 39).

Recently, ML algorithms have played an important role in predicting diseases (40). ML models, a branch of artificial intelligence, utilize mathematical algorithms to identify and classify patterns in diverse datasets for decision-making. While these models exhibit high performance, their lack of transparency is a significant drawback. This opacity, often described as a “black-box” issue, becomes particularly problematic in clinical settings. Despite their high accuracy, the inability to comprehend the rationale behind the model's predictions causes apprehension among clinicians relying on these predictions for treatment or prevention, thereby impeding the broader application of ML models (41, 42). In this research, we developed explainable ML models and combined with the DALEX package to improve the user's understanding of how decisions are made within an ML model, thereby increasing its transparency and reliability. Our model is adept at identifying patients with a high risk of IS, enabling the strategic prioritization of scarce medical resources for those in critical need and thus streamlining the distribution of these resources.

This study has several limitations. First, the duration of heavy metal exposure is unknown, limiting our ability to assess long-term effects. We also did not disaggregate race, age, or other characteristics due to computational constraints, potentially affecting the specifics of our subgroup analyses. Being a retrospective study, it inevitably carries bias. Specifically, the NHANES data include self-reported information, which may introduce recall bias and inaccuracies. In addition, while the NHANES strives for representativeness, there is still a possibility of selection bias. Regarding the ML models, there is a risk of overfitting despite using a cross-validation technique. The absence of certain confounding variables in the NHANES dataset may also introduce bias. Future research should consider these factors and aim for validation across different populations.

## 5 Conclusion

In this study, we suggest that LR and XGBoost models can be reliable tools for identifying patients at a high risk of IS. Factors such as age, total mercury in blood, PIR, and cadmium levels were significantly related to IS.

## Data Availability

The original contributions presented in the study are included in the article/Supplementary material, further inquiries can be directed to the corresponding author.

## Supplementary material

Supplementary Figure 1 The precision-recall curve for the seven ML models, including RF, XGBoost, LightGBM, DT, LR, kNN, and DT.

Supplementary Figure 2 The confusion matrix on the test data for the seven ML models, including RF, XGBoost, LightGBM, DT, LR, kNN, and DT.

## References

[1] Ajoolabady A Wang S Kroemer G Penninger JM Uversky VN Pratico D. Targeting autophagy in ischemic stroke: from molecular mechanisms to clinical therapeutics. Pharmacol Ther. (2021) 225:107848. 10.1016/j.pharmthera.2021.107848 33823204 PMC8263472

[2] Ferrari F Villa RF. Brain bioenergetics in chronic hypertension: risk factor for acute ischemic stroke. Biochem Pharmacol. (2022) 205:115260. 10.1016/j.bcp.2022.115260 36179931

[3] Zhang L Li X Wolfe CDA O'Connell MDL Wang Y. Diabetes as an independent risk factor for stroke recurrence in ischemic stroke patients: an updated meta-analysis. Neuroepidemiology. (2021) 55:427–35. 10.1159/000519327 34673640

[4] Welten S Onland-Moret NC Boer JMA Verschuren WMM van der Schouw YT. Age at menopause and risk of ischemic and hemorrhagic stroke. Stroke. (2021) 52:2583–91. 10.1161/STROKEAHA.120.030558 34078111 PMC8312566

[5] Lai SM Alter M Friday G Sobel E. A multifactorial analysis of risk factors for recurrence of ischemic stroke. Stroke. (1994) 25:958–62. 10.1161/01.STR.25.5.958 8165690

[6] Medina-Estévez F Zumbado M Luzardo OP Rodríguez-Hernández Á Boada LD Fernández-Fuertes F. Association between heavy metals and rare earth elements with acute ischemic stroke: a case-control study conducted in the Canary Islands (Spain). Toxics. (2020) 8:66. 10.3390/toxics8030066 32887274 PMC7560340

[7] Yen CC Chen HH Hsu YT Tseng CJ Lin CH. Effects of heavy metals in acute ischemic stroke patients: a cross-sectional study. Medicine (Baltimore). (2022) 101:e28973. 10.1097/MD.0000000000028973 35244065 PMC8896421

[8] Mattern L Chen C McClure LA Brockman J Cushman M Judd S. Serum zinc levels and incidence of ischemic stroke: the reasons for geographic and racial differences in stroke study. Stroke. (2021) 52:3953–60. 10.1161/STROKEAHA.120.033187 34412513 PMC8608709

[9] Hussain M Mumtaz S. E-waste: impacts, issues and management strategies. Rev. Environ. Health (2014) 29:53–8. 10.1515/reveh-2014-0016 24695030

[10] Handelman GS Kok HK Chandra RV Razavi AH Lee MJ Asadi H. eDoctor: machine learning and the future of medicine. J Intern Med. (2018) 284:603–19. 10.1111/joim.12822 30102808

[11] Alber M Buganza Tepole A Cannon WR De S Dura-Bernal S Garikipati K Karniadakis G. Integrating machine learning and multiscale modeling-perspectives, challenges, and opportunities in the biological, biomedical, and behavioral sciences. NPJ Digit Med. (2019) 2:115. 10.1038/s41746-019-0193-y 31799423 PMC6877584

[12] Zhang B Dong X Hu Y Jiang X Li G. Classification and prediction of spinal disease based on the SMOTE-RFE-XGBoost model. PeerJ Comput Sci. (2023) 9:e1280. 10.7717/peerj-cs.1280 37346612 PMC10280425

[13] Liu J Li X Zhu P. Effects of various heavy metal exposures on insulin resistance in non-diabetic populations: interpretability analysis from machine learning modeling perspective. Biol Trace Elem Res. (2024). 10.1007/s12011-024-04126-3 38409445

[14] Tachie CYE Obiri-Ananey D Tawiah NA-O Attoh-Okine N Aryee AA-O. Machine learning approaches for predicting fatty acid classes in popular US snacks using NHANES data. Nutrients (2023) 15:3310. 10.3390/nu15153310 37571247 PMC10421424

[15] Carrington AA-O Fieguth PW Qazi H Holzinger A Chen HH Mayr F. A new concordant partial AUC and partial c statistic for imbalanced data in the evaluation of machine learning algorithms. BMC Med Inform Decis Mak. (2020) 20:4. 10.1186/s12911-019-1014-6 31906931 PMC6945414

[16] Liu W Wang S Ye Z Xu P Xia X Guo MA-O. Prediction of lung metastases in thyroid cancer using machine learning based on SEER database. Cancer Med. (2022) 11:2503–15. 10.1002/cam4.4617 35191613 PMC9189456

[17] Chicco D Jurman G. The Matthews correlation coefficient (MCC) should replace the ROC AUC as the standard metric for assessing binary classification. BioData Min. (2023) 16:4. 10.1186/s13040-023-00322-4 36800973 PMC9938573

[18] Angraal S Mortazavi BJ Gupta A Khera R Ahmad T Desai NR. Machine learning prediction of mortality and hospitalization in heart failure with preserved ejection fraction. JACC: Heart Fail. (2020) 8:12–21. 10.1016/j.jchf.2019.06.013 31606361

[19] Zheng Y Wang J Ling Z Zhang J Zeng Y Wang K. A diagnostic model for sepsis-induced acute lung injury using a consensus machine learning approach and its therapeutic implications. J Transl Med. (2023) 21:620. 10.1186/s12967-023-04499-4 37700323 PMC10498641

[20] Mozaffarian D Benjamin EJ Go AS Arnett DK Blaha MJ Cushman M. Heart Disease and Stroke Statistics-2016 update: a report from the american heart association. Circulation. (2016) 133:e38–360. 26673558 10.1161/CIR.0000000000000350

[21] Liu L Wang D Wong KS Wang Y. Stroke and stroke care in China: huge burden, significant workload, and a national priority. Stroke. (2011) 42:3651–4. 10.1161/STROKEAHA.111.635755 22052510

[22] Boehme AK Esenwa C Elkind MS. Stroke risk factors, genetics, and prevention. Circ Res. (2017) 120:472–95. 10.1161/CIRCRESAHA.116.308398 28154098 PMC5321635

[23] Lin CH Hsu YT Yen CC Chen HH Tseng CJ Lo YK. Association between heavy metal levels and acute ischemic stroke. J Biomed Sci. (2018) 25:49. 10.1186/s12929-018-0446-0 29801491 PMC5970463

[24] Tansel B. From electronic consumer products to e-wastes: global outlook, waste quantities, recycling challenges. Environ Int. (2017) 98:35–45. 10.1016/j.envint.2016.10.002 27726897

[25] Moon KA Oberoi S Barchowsky A Chen Y Guallar E Nachman KE. A dose-response meta-analysis of chronic arsenic exposure and incident cardiovascular disease. Int J Epidemiol. (2018) 47:1013. 10.1093/ije/dyy073 29697784 PMC6005049

[26] Navas-Acien A Selvin E Sharrett AR Calderon-Aranda E Silbergeld E Guallar E. Lead, cadmium, smoking, and increased risk of peripheral arterial disease. Circulation. (2004) 109:3196–201. 10.1161/01.CIR.0000130848.18636.B2 15184277

[27] Houston MC. Role of mercury toxicity in hypertension, cardiovascular disease, and stroke. J Clin Hypertens (Greenwich). (2011) 13:621–7. 10.1111/j.1751-7176.2011.00489.x 21806773 PMC8108748

[28] Yoshizawa K Rimm EB Morris JS Spate VL Hsieh CC Spiegelman D. Mercury and the risk of coronary heart disease in men. N Engl J Med. (2002) 347:1755–60. 10.1056/NEJMoa021437 12456851

[29] Wiggers GA Peçanha FM Briones AM Pérez-Girón JV Miguel M Vassallo DV. Low mercury concentrations cause oxidative stress and endothelial dysfunction in conductance and resistance arteries. Am J Physiol Heart Circ Physiol. (2008) 295:H1033–h1043. 10.1152/ajpheart.00430.2008 18599595

[30] Salonen JT Seppänen K Nyyssönen K Korpela H Kauhanen J Kantola M. Intake of mercury from fish, lipid peroxidation, and the risk of myocardial infarction and coronary, cardiovascular, and any death in eastern Finnish men. Circulation. (1995) 91:645–55. 10.1161/01.CIR.91.3.645 7828289

[31] Chiou HY Huang WI Su CL Chang SF Hsu YH Chen CJ. Dose-response relationship between prevalence of cerebrovascular disease and ingested inorganic arsenic. Stroke. (1997) 28:1717–23. 10.1161/01.STR.28.9.1717 9303014

[32] Steenland K Barry V Anttila A Sallmén M McElvenny D Todd AC. A cohort mortality study of lead-exposed workers in the USA, Finland and the UK. Occup Environ Med. (2017) 74:785–91. 10.1136/oemed-2017-104311 28546320

[33] Tsinovoi CL Xun P McClure LA Carioni VMO Brockman JD Cai J. Arsenic exposure in relation to ischemic stroke: the reasons for geographic and racial differences in stroke study. Stroke. (2018) 49:19–26. 10.1161/STROKEAHA.117.018891 29212736 PMC5742041

[34] Gaman L Radoi MP Delia CE Luzardo OP Zumbado M Rodríguez-Hernández Á. Concentration of heavy metals and rare earth elements in patients with brain tumours: analysis in tumour tissue, non-tumour tissue, and blood. Int J Environ Health Res. (2021) 31:741–54. 10.1080/09603123.2019.1685079 31674203

[35] Hammond D O'Connor RJ. Constituents in tobacco and smoke emissions from Canadian cigarettes. Tob Control. (2008) 17:i24–31. 10.1136/tc.2008.024778 18768456

[36] Nawrot TS Van Hecke E Thijs L Richart T Kuznetsova T Jin Y. Cadmium-related mortality and long-term secular trends in the cadmium body burden of an environmentally exposed population. Environ Health Perspect. (2008) 116:1620–8. 10.1289/ehp.11667 19079711 PMC2599754

[37] Tinkov AA Filippini T Ajsuvakova OP Skalnaya MG Aaseth J Bjørklund G. Cadmium and atherosclerosis: a review of toxicological mechanisms and a meta-analysis of epidemiologic studies. Environ Res. (2018) 162:240–60. 10.1016/j.envres.2018.01.008fpubh-12-1388257 29358116

[38] Wen Y Huang S Zhang Y Zhang H Zhou L Li D. Associations of multiple plasma metals with the risk of ischemic stroke: a case-control study. Environ Int. (2019) 125:125–34. 10.1016/j.envint.2018.12.037 30716572

[39] Peters JL Perlstein TS Perry MJ McNeely E Weuve J. Cadmium exposure in association with history of stroke and heart failure. Environ Res. (2010) 110:199–206. 10.1016/j.envres.2009.12.004 20060521 PMC3031174

[40] Akyea RK Qureshi N Kai J Weng SF. Performance and clinical utility of supervised machine-learning approaches in detecting familial hypercholesterolaemia in primary care. NPJ Digit Med. (2020) 3:142. 10.1038/s41746-020-00349-5 33145438 PMC7603302

[41] Cohen IG Amarasingham R Shah A Xie B Lo B. The legal and ethical concerns that arise from using complex predictive analytics in health care. Health Aff (Millwood). (2014) 33:1139–47. 10.1377/hlthaff.2014.0048 25006139

[42] Guan C Ma F Chang S Zhang J. Interpretable machine learning models for predicting venous thromboembolism in the intensive care unit: an analysis based on data from 207 centers. Crit Care. (2023) 27:406. 10.1186/s13054-023-04683-4 37875995 PMC10598960

