# Supplementary figures and images for: Machine learning approaches to identify the link between heavy metal exposure and ischemic stroke using the US NHANES data from 2003 to 2018
Source: Front Public Health. 2024 Sep 16;12:1388257. doi: 10.3389/fpubh.2024.1388257 (PMC11439780; doi:10.3389/fpubh.2024.1388257)

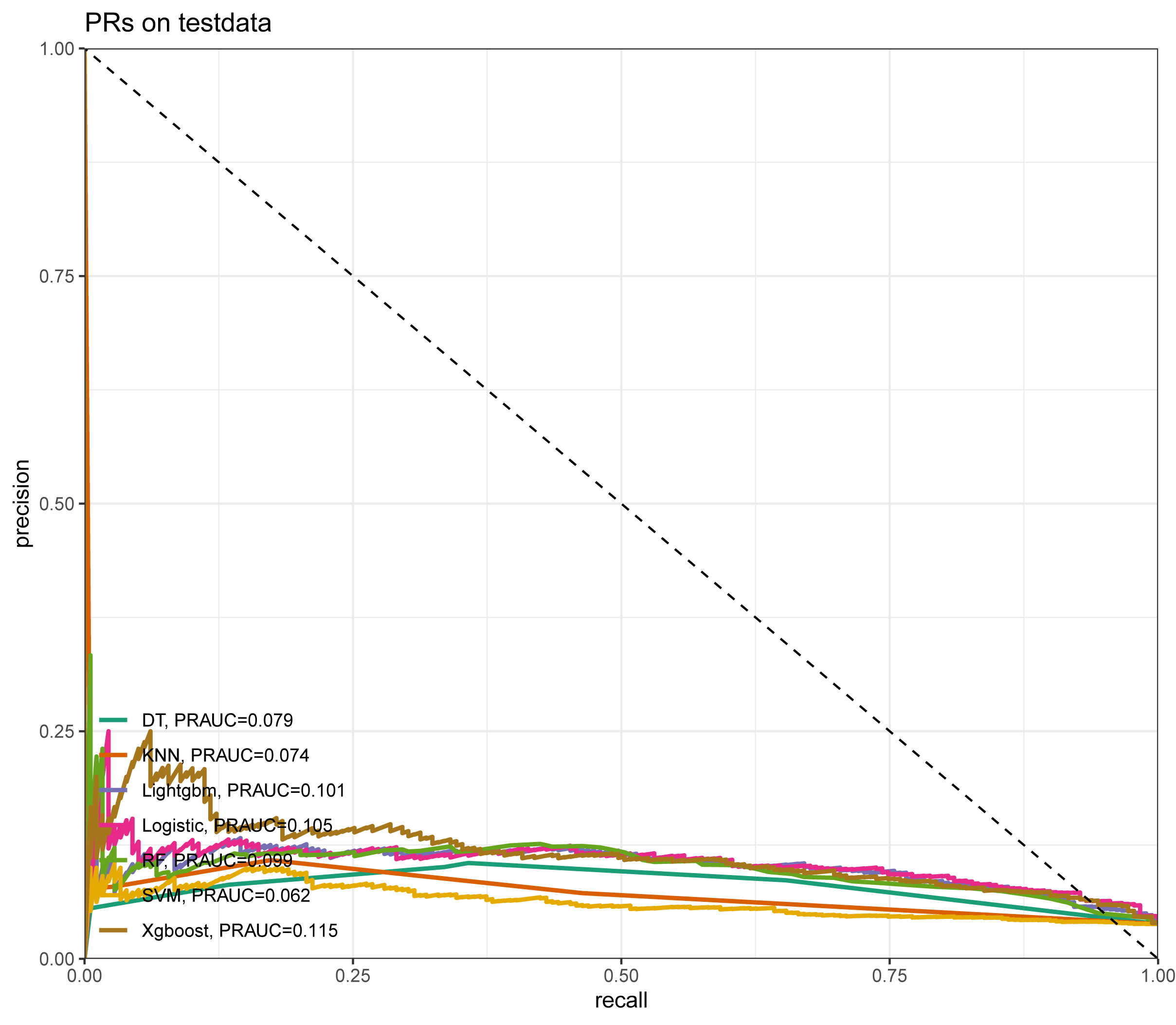

Supplement: Supplementary Figure 1 — The precision-recall curve for the seven ML models, including RF, XGBoost, LightGBM, DT, LR, kNN, and DT. [file Image_1.TIF]

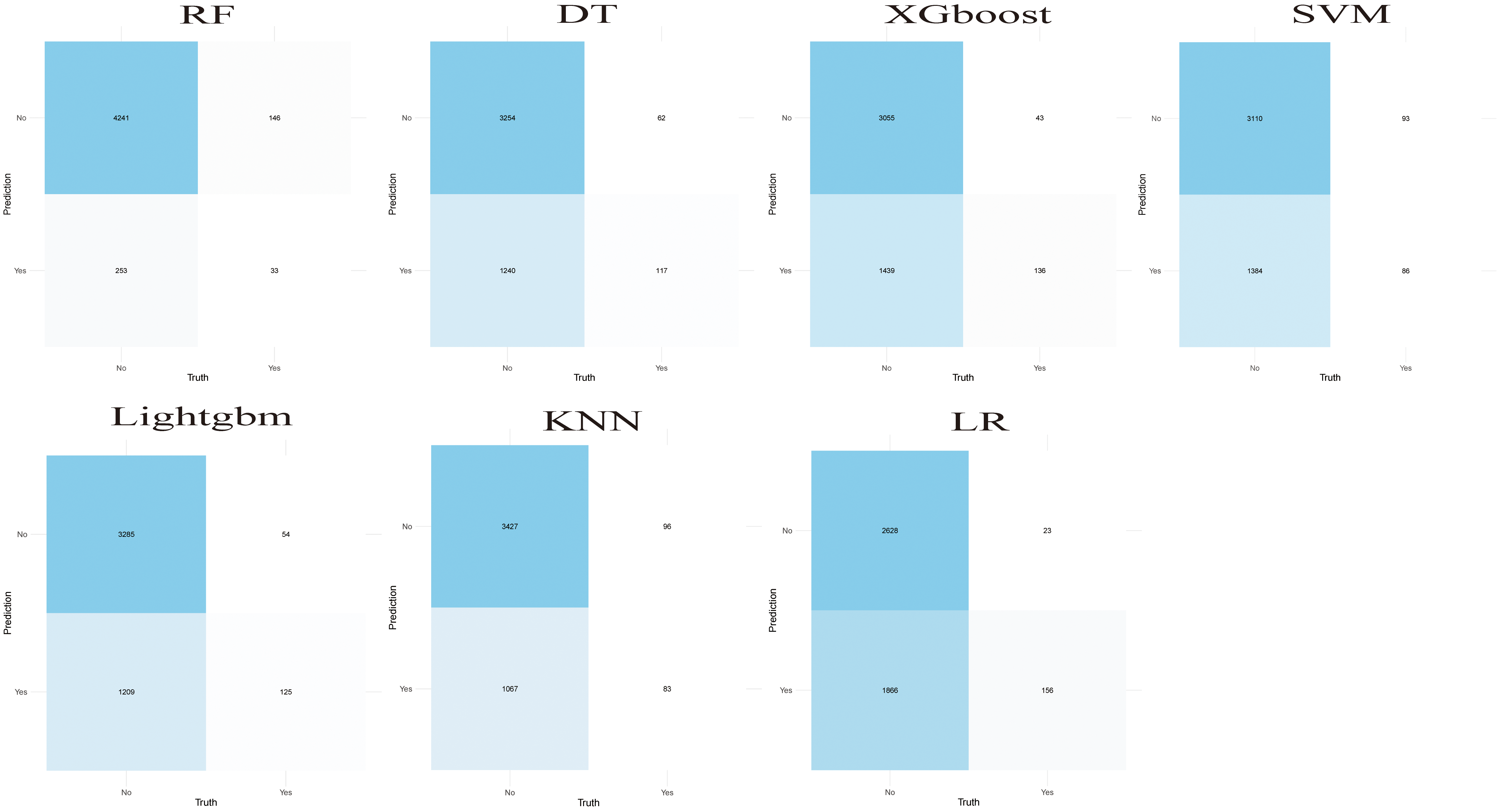

Supplement: Supplementary Figure 2 — The confusion matrix on the test data for the seven ML models, including RF, XGBoost, LightGBM, DT, LR, kNN, and DT. [file Image_2.TIF]
